# Supplementary material for: Observation of a mixed close-packed structure in superionic water
Source: Nat Commun. 2025 Dec 7;17:374. doi: 10.1038/s41467-025-67063-2 (PMC12796318; doi:10.1038/s41467-025-67063-2)
Supplement: Supplementary file 1 — Supplementary Information [file 41467_2025_67063_MOESM1_ESM.pdf]

Supplementary Information
Observation of a mixed close-packed structure in superionic water

L. Andriambariarijaona*,[1] M. G. Stevenson*,[2] M. Bethkenhagen,[1] L. Lecherbourg,[3,4] F. Lefèvre,[1] T. Vinci,[1] K. Appel,[5] C. Baehtz,[6] A. Benuzzi-Mounaix,[1] A. Bergermann,[7] D. Bespalov,[5] E. Brambrink,[5] T. E. Cowan,[6] E. Cunningham,[7] A. Descamps,[8] S. Di Dio Cafiso,[6] G. Dyer,[7] L. B. Fletcher,[7] M. French,[2] M. Frost,[7] E. Galtier,[7] A. E. Gleason,[7] S. H. Glenzer,[7] G. D. Glenn,[7,9] Y. Guarnelli,[10] N. J. Hartley,[7] Z. He,[2] M.-L. Herbert,[2,6] J.-A. Hernandez,[11] B. Heuser,[2,6] H. Höppner,[6] O. S. Humphries,[6,5] R. Husband,[12] D. Khaghani,[7] Z. Konôpková,[5] J. Kuhlke,[2,6] A. Laso Garcia,[6] H. J. Lee,[7] B. Lindqvist,[2,6] J. Lütgert,[2] W. Lynn,[8] M. Masruri,[6] P. May,[2] E. E. McBride,[8] B. Nagler,[7] M. Nakatsutsumi,[5] J.-P. Naedler,[2] B. K. Ofori-Okai,[7] S. Pandolfi,[10] A. Pelka,[6] T. R. Preston,[5] C. Qu,[2] L. Randolph,[5] D. Ranjan,[2,6,12] R. Redmer,[2] J. Rips,[2] C. Schoenwaelder,[7] S. Schumacher,[2] A. K. Schuster,[6] J.-P. Schwinkendorf,[6,5] C. Strohm,[12] M. Tang,[5,12] T. Toncian,[6] K. Voigt,[6] J. Vorberger,[6] U. Zastrau,[5] D. Kraus,[2,6] and A. Ravasio[1]

[1] *Laboratoire LULI, CNRS – École Polytechnique – CEA – Sorbonne Université, 91128 Palaiseau, France*
[2] *Institut für Physik, Universität Rostock, Albert-Einstein-Str. 23, 18059 Rostock, Germany*
[3] *CEA, DAM, DIF, 91297 Arpajon, France*
[4] *Laboratoire Matière en Conditions Extrêmes, Université Paris-Saclay, CEA, 91680 Bruyères-le-Châtel, France*
[5] *European XFEL, Holzkoppel 4, 22869 Schenefeld, Germany*
[6] *Helmholtz-Zentrum Dresden-Rossendorf, Bautzner Landstr. 400, 01328 Dresden, Germany*
[7] *SLAC National Accelerator Laboratory, Menlo Park, CA 94025, USA*
[8] *School of Mathematics and Physics, Queen's University Belfast, University Road, Belfast BT7 1NN, UK*
[9] *Department of Applied Physics, Stanford University, Stanford, CA 95064, USA*
[10] *Institut de Minéralogie, Physique des Matériaux et Cosmochimie (IMPMC), Sorbonne Université, MNHN, CNRS UMR 7590, 75005 Paris, France*
[11] *European Synchrotron Radiation Facility (ESRF), Grenoble, France*
[12] *Deutsches Elektronen-Synchrotron DESY, Notkestr. 85, 22607 Hamburg, Germany*

Supplementary Contents

1. Velocimetry

2. Hydrodynamic simulations

3. Estimation of the thermodynamic conditions

4. X-ray diffraction and data analysis

4.1 Diffraction signal from diamond
4.2 Observation of body-centered cubic
4.3 Observation of BCC + FCC coexistence

5. Refinement for stacking fault

6. Ruling out other possible interpretations of the SF data

6.1 FCC only phase
6.2 Mixture of FCC + BCC structures
6.3 Diamond contribution
6.4 Liquid contribution
6.5 Grain size broadening

7. H_2O high-pressure diagram

1. VELOCIMETRY

Velocimetry data collected with two Velocity Interferometer from Any Reflector (VISAR) systems [1], were used to track the velocities of various interfaces, providing constraints on the hydrodynamics of reverberating shocks. These constraints allowed us to determine the thermodynamic conditions reached in the target at the time of X-ray probing through hydrodynamic simulations. While the diamond ablator always had both faces coated with 100 nm of Al, different coating configurations were used for the rear diamond window, enabling access to different interface velocities.

In some cases (Fig.2 and Fig. S1a), half of the rear-side diamond face in contact with water was coated with 100 nm of Al. Under these conditions, and while the water remained transparent during compression, we were able to track the diamond ablator/water interface and determine its apparent velocity. When the shock broke out from the target, we also tracked the free surface velocity (u_f) of the rear diamond window (Fig. S1a and b). In a few shots, we could also follow the water/diamond window interface through the diamond window itself (Fig. S1c). In these cases, the real particle velocity was retrieved from measured apparent velocity accounting for the diamond refractive index as a function of compression based on [2]. During the experimental campaign at the EuXFEL, the VISAR data quality was unfortunately insufficient to directly infer velocities. Instead, we calibrated the simulations using the shock arrival in water together with the entrance and exit times of the diamond window. (Fig. S1d)

2. HYDRODYNAMIC SIMULATIONS

To get a reliable reconstruction of the material's compression history from the velocimetry data, we employed the one-dimensional Lagrangian radiative hydrodynamic Esther code [3]. The simulated targets replicate the real experimental setup, consisting of a few 100 nm aluminium layer, 30 µm of diamond, a water layer between 30 and 60 µm thick, followed by another 60 μm of diamond. The initial aluminium layer ensures energy deposition occurs at the front of the target. Laser propagation is modelled by solving the Helmholtz equation in each cell. While aluminium layers (few 100 nm) are present at the diamond-water interfaces in the real target, they are not included in the simulation, as they have minimal influence on the results. Additionally, simulations indicate that radiation effects are negligible. We aim to employ the most precise Equation of state (EOS) available to date. For aluminium, we used the SESAME 3720 EOS table, while for diamond, we used the SESAME 7830 EOS table. The strength of diamond is modelled using a perfectly plastic constitutive framework, in which both the elastic yield strength and shear modulus are treated as constants within the solid phase. These values drop to zero when the diamond reaches its melting temperature. For the simulations, the elastic yield strength and shear modulus are set to 80 GPa and 534 GPa, respectively [4]. For water, we use the recently developed AQUA EOS [5], which includes *ab initio* results in the high-pressure region [6, 7]. These data lead to a much improved description of the ice phases VII/VII′/X, the superionic region and dense plasmas [8, 9]. A comparison with the widely used SESAME EOS model [10] highlights significant discrepancies, particularly in the high-pressure region, where the SESAME EOS relies on a Thomas-Fermi model.

Fig. S2 highlights the differences between the AQUA and the SESAME 7154 EOS throughout the available density-temperature plane. The most severe discrepancies can be found at low densities (below 1 g/cm^3), nevertheless, we find the impact of the EOS choice to be significant in the region relevant for our experiment as illustrated exemplary in Fig. S3 for the principle hugoniot starting from 1 g/cm^3, 1 bar and 295 K. One of the most notable improvements is the temperature estimation, which now aligns much more closely with ab initio predictions [11, 12].

To interpret the VISAR data, for each shot we run optimised hydrodynamic simulations. The initial estimates were based on the measured drive-laser pulse shape and theoretical intensity. The shock arrival timings at the diamond window interface and its breakout, along with the apparent (when available) and free surface velocities, served as strict constraints for determining the laser intensity and refining the target thickness (Fig. S1a,b,c,d). Inferring the true velocity of the diamond ablator/water interface in this transparent regime requires knowledge of the compression history as well as the optical properties of compressed water—factors that are difficult to extract solely from VISAR data. Instead, we calculated the corresponding apparent velocity in the simulations estimating at each time-step the optical path (e.g., [13]) and using the water refractive index as a function of density from Ref [14]. The VISAR-measured apparent velocity history was then used to constrain the simulations.
For shots in which the entire diamond window was coated, the apparent velocity at the diamond ablator/water interface was inaccessible. However, shock timing, free surface velocity, or interface velocity at the water/rear diamond window interface were measured and used to optimise the simulations. Laser intensity and target thickness were iteratively adjusted within their uncertainties to match the VISAR data (Fig. S1a,b,c,d).
For the EuXFEL data, simulations were primarily constrained by the shock arrival timing at the diamond windows and the shock breakout (Fig. S1d). However, the high reproducibility of the DIPOLE laser provided additional help in refining the laser intensities.

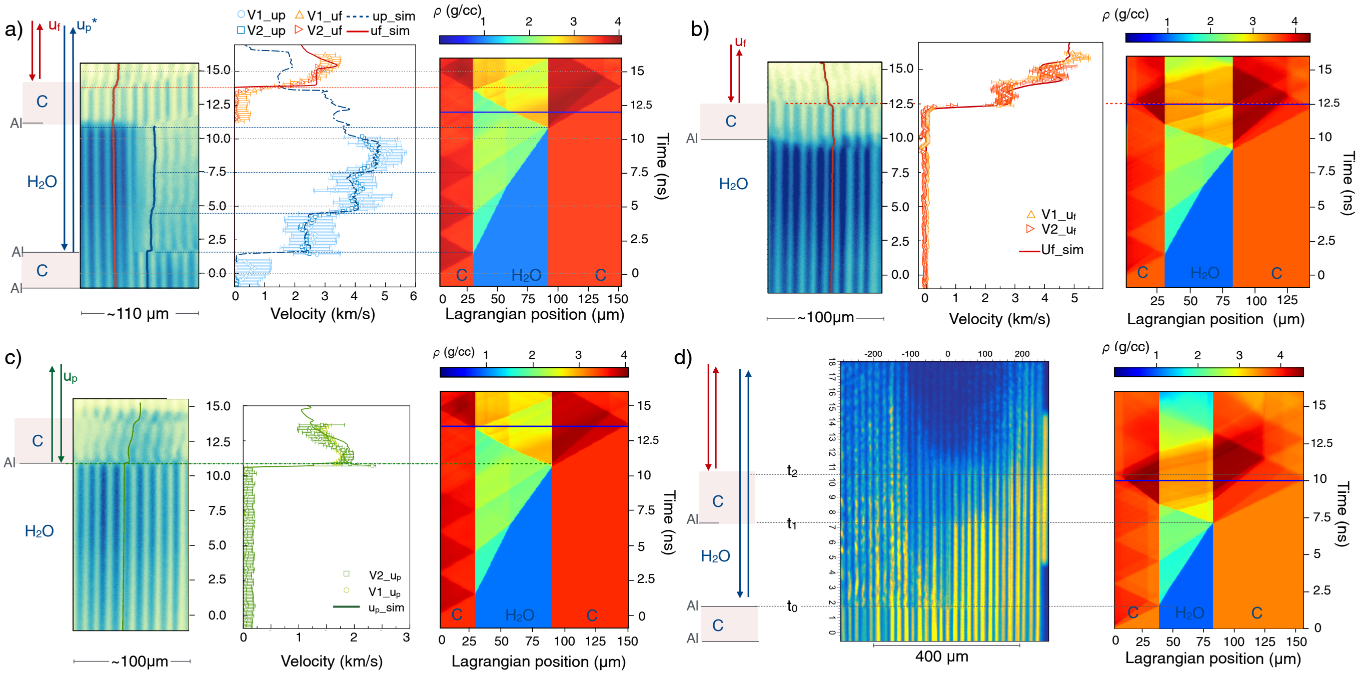

FIG. S1. VISAR data and hydrodynamic simulations as in Fig. 2 but for different shots with distinct target configurations. Uncertainties in the velocity measurements correspond to the standard deviation of fringe-shift values within the analyzed region. a) LCLS Run 233 has the same target configuration described in Fig. 2. b) In LCLS Run 250 we only measure free surface velocity. c) For LCLS Run 239 we can measure the water/diamond window interface velocity. d) For EuXFEL Run 111 we only detect the shock arrival in water together with the entrance and exit times of the diamond window. These information are used to calibrate hydrodynamic simulations done with Esther code and get the thermodynamic conditions at the probing times (blue line in the simulation density map).

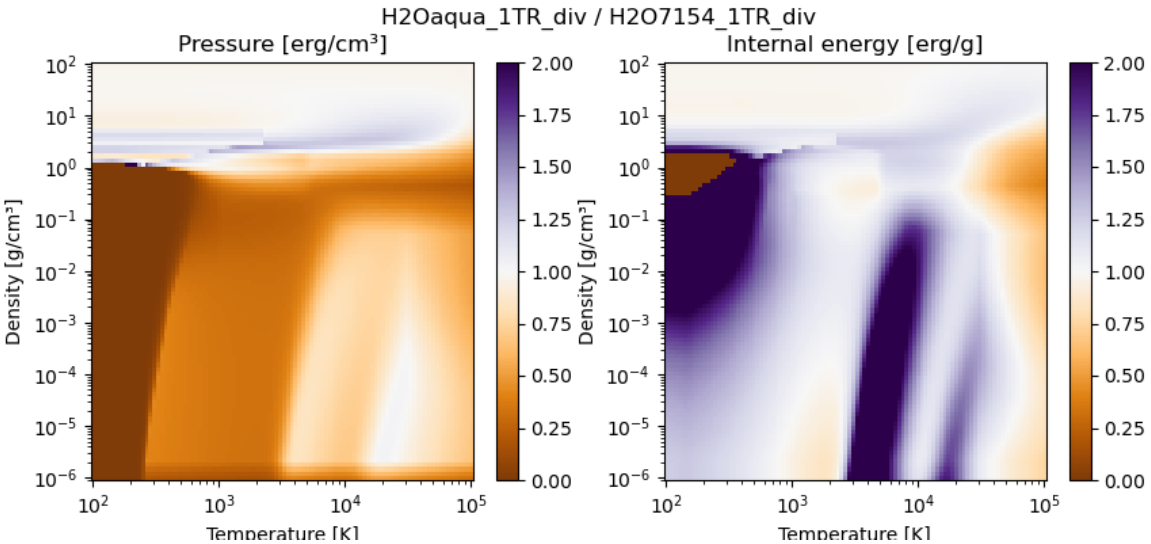

FIG. S2. Difference between the AQUA and SESAME 7154 EOS in pressure (left panel) and internal energy (right panel).

3. ESTIMATION OF THE THERMODYNAMIC CONDITIONS

Once a satisfactory agreement was achieved between simulations and velocimetry data, the density, pressure, and temperature conditions in the water layer were extracted from the corresponding simulations. Given that the probing time (<50 fs) was significantly shorter than the typical hydrodynamic timescale (∼few 10 ps), the thermodynamic profiles could be considered instantaneous (blue lines in the density maps on Fig. S1).
At LCLS, the targets were ∼50 µm thick and this thick-

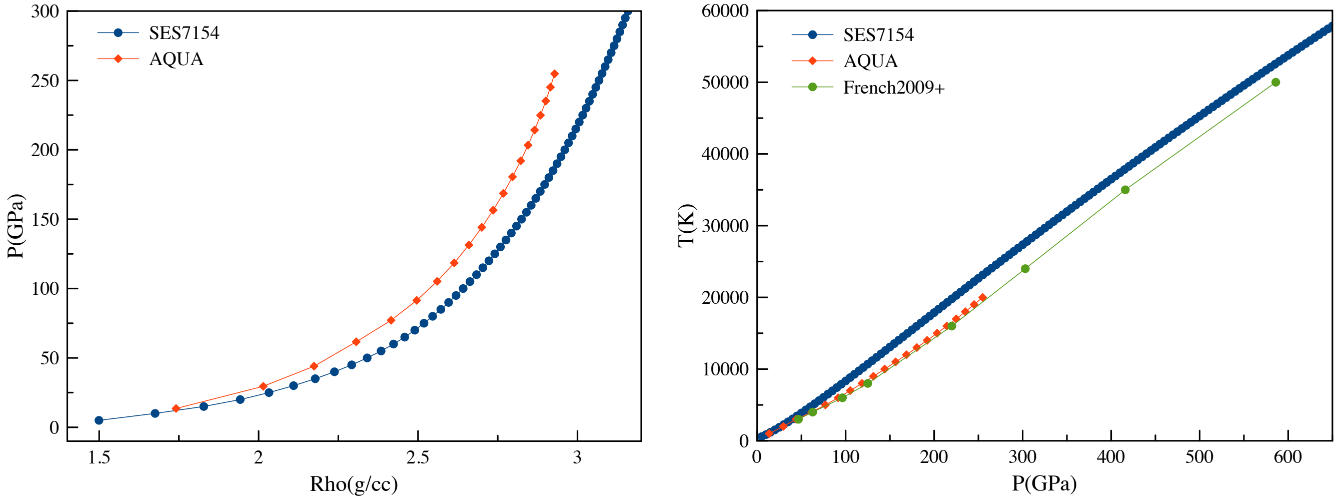

FIG. S3. Comparison of the principle Hugoniot curve derived from AQUA (red) [5] and SESAME 7154 (blue) [10] for pressure/density (left panel) and temperature/pressure (right panel). Temperatures predicted with AQUA are in very good agreement with ab initio calculations (green) [11, 12].

ness did not align with the timing of the reverberations of the elastic precursor in the 30 µm diamond ablator. As a result, a spatial variation in compression developed in the target due to the first wave being overtaken by the second reverberation within the water layer. This led to a steep temperature gradient, with part of the target experiencing one fewer compression cycle and reached a higher temperature with respect of the first region of the sample, where the additional compression lower the reached temperature. (Fig. S4a,b).

The contribution of the high-temperature part (yellow area in Fig. S4a,b) of the target was mainly found to be in the form of a liquid background, instead of crystalline phases. This aligns with the observation that, at all probing times, simulations suggest conditions close to melting or above (Fig. S4a,b and Fig. S18). The main contribution to the XRD signal comes from the colder part of the target, where quite uniform conditions are found (blue area in Fig. S4a,b). In line with our interpretations, for these shots, the refinement necessitates to account for a large amount of liquid background (section 4). This is different from the EuXFEL data (see Fig. S7 and Fig. S8, where no liquid background is required to fit the diffraction patterns. Indeed, in the experiment at the EuXFEL, the targets were much thinner (∼30 µm) and better dimensioned to match the reverberations in the diamond ablator. In this case the compression history and gradients are substantially different from those obtained at LCLS and in general, very uniform conditions are generated (see Fig. S4c,d). Regardless the different compression histories, we found that for similar XRD data, close hydrodynamic conditions for the cold part of the LCLS target and the EuXFEL shots are predicted (see Fig. S4, Fig. S7,Fig. S8 and Table S1). This corroborates our estimation of the thermodynamic conditions. Error bars are determined considering different simulations giving agreement within the VISAR error bars.

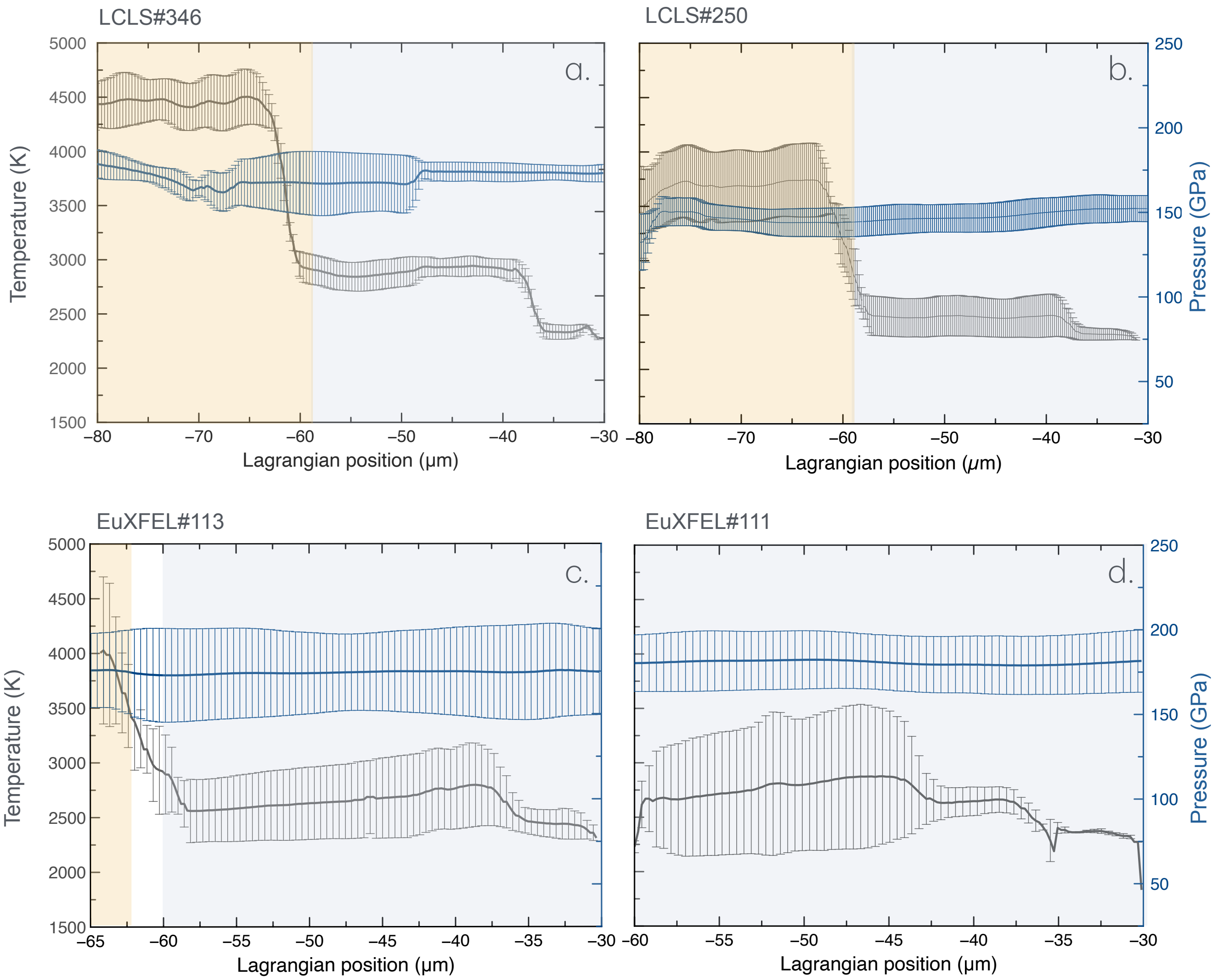

FIG. S4. Temperature (gray) and Pressure (blue) profiles in the water layer at the x-ray probing times for similar shots at LCLS (a,b) and EuXFEL (c,d). Mean conditions and uncertainties are obtained from hydrodynamic simulations consistent within VISAR error bars. Yellow shadow corresponds to the part of the target undergoing one fewer compression cycle and hence experiencing higher temperatures. Blue shadow corresponds to the target region contributing to the crystalline features in the diffraction data.

4. X-RAY DIFFRACTION AND DATA ANALYSIS

The *in situ* XRD was performed using 9.5 and 18 keV x-rays from the Free Electron Lasers at the LCLS and Eu-XFEL respectively. The XRD data were collected using the ePIX detectors at the MEC in a standard configuration and the Varex twin detectors at the EuXFEL.

In both campaigns, to calibrate the sample-to-detector distance, detector tilts, and determine instrumental broadening, we used a NIST Standard Reference Material CeO_2 (EuXFEL) and LaB_6 (LCLS) powder. An example of the Rietveld refinement of CeO_2 is shown in Fig. S5. On the basis of the refinement results, the instrumental resolution function (IRF) is determined, which was used for the refinement of all experimental data. A one-dimensional azimuthal-integrated diffracted intensities as a function of the scattering vector Q were obtained using Dioptas software [15]. This stage involved combining images from different detector modules, such as VAREX I and II for the EuXFEL, and Quads 2 and 3 for the LCLS (see Fig. S7 and Fig. S8).

Using the FullProf software [16], we performed Le Bail refinements on integrated diffraction data; examples under different P–T conditions are shown in Fig. S7, S8 and in the main text, Fig. 3.
For some datasets, particularly those from LCLS, where the sample thickness was significant, a contribution from the liquid phase was observed. To account for this contribution to the refinement, the background was modelled using the diffraction pattern of a liquid structure obtained from *ab initio* calculations (see section 6 6.4), as illustrated in Fig. S8a. For the very high-pressure data, where stacking faults were observed, additional refinements were carried out using the DIFFAX and FAULTS software packages [17, 18] (see section 5).
For each shot, the refinement provided the unit cell volume (V) and the corresponding density (ρ) for each phase, as reported in Table S1. For each observed phase, the uncertainty in volume (V_{err}) was estimated from the standard deviation of the volumes determined from individual reflections. The uncertainty in density was calculated using standard error propagation.

4.1. Diffraction signal from diamond

The collected XRD signal is associated with the different diffracting components of the target assembly: the water sample, the diamond ablator, and the diamond windows. To minimise the diamond contributions to the overall diffraction signal, single-crystal diamonds were mainly used. In the presented data-set only three among the shown data comprised polycrystalline diamonds: LCLS Run 233 and LCLS Run 119, with both polycrystalline ablator and windows, and LCLS Run 346, with a polycrystalline ablator and a single crystal window. X-ray-only data were taken prior to the laser shot.

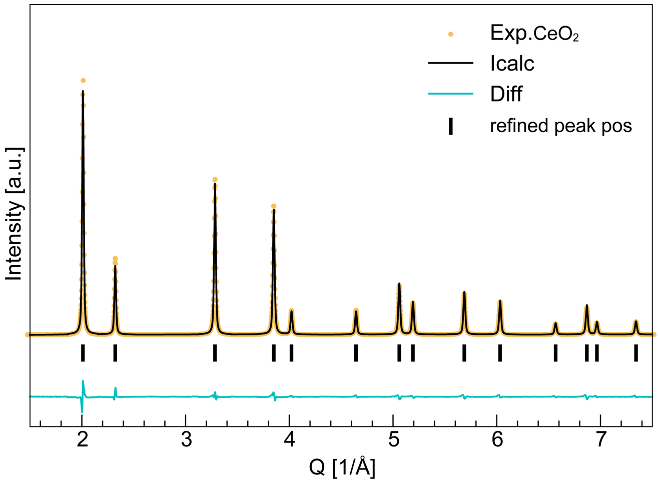

FIG. S5. Rietveld refinement of the CeO_2 standard was performed to calibrate the sample-to-detector distance, correct for detector tilts, and determine the instrumental broadening. A Thompson–Cox–Hastings pseudo-Voigt peak shape function, incorporating axial divergence asymmetry, was used to model the Bragg reflections. The refined shape function were $U = 0.0, V = 0.012011, W = 0.004258, X = 0.058403, Y = 0.057187, Z_{\mathrm{zero}} = -0.0026$. Using the Caglioti function, defined as $\mathrm{FWHM}^2 = U \cdot \tan^2\theta + V \cdot \tan\theta + W$, the instrumental broadening was extracted as a function of the diffraction angle. These parameters were stored in IRF files and systematically applied in all our refinement.

They show no significant diffraction contribution from the single-crystal samples and a stronger "spotty" contribution in the case of polycrystalline diamonds (see Fig. S6). In both cases, the contribution of liquid water at lower angles is visible.

Under shock compression, also single-crystal diamonds may fragment into multiple crystallites, resulting in a textured and localised diffraction pattern that contrasts with the powder-like diffraction lines typical of polycrystalline high-pressure ice from compressed water. This can be seen in several shots, for example in Fig. S7.
The diamond diffraction can be very intense and at some conditions might overlap with the water main diffraction peaks. However, in those cases, such contribution can be easily masked before integration or excluded during refinement, as it is limited in the $\phi - Q$ space (see Fig. S7a). Owing to the pronounced difference between the diffraction signal from diamond and that of polycrystalline H_2O powder (Fig. S7), there is no ambiguity in identifying the peaks assignment.

4.2. Observation of body-centered cubic

BCC phases were observed in data collected during Eu-XFEL run 112 and LCLS run 119 and 233 (see Table S1). Fig. S7a,b shows the raw data together with both the integrated intensities and the corresponding Le Bail refinements for runs 233 and 119, while the same for run 112

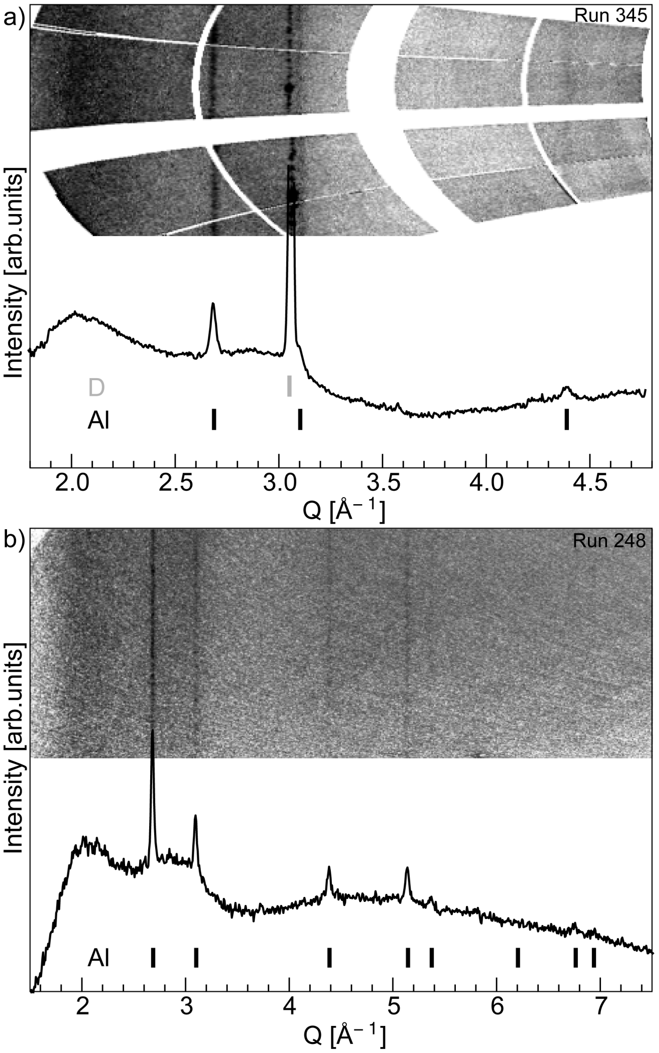

FIG. S6. X-ray diffraction patterns from targets equipped with polycrystalline (a) and single-crystal (b) diamonds collected from un-driven targets at LCLS and EuXFEL. The corresponding 2D diffraction images are shown as insets. Vertical markers indicate the Bragg positions of aluminium (black) and diamond (grey). In (a) a strong diffraction signal from the polycrystalline diamond is observed. In contrast, no significant diffraction contribution is detected from the single-crystal diamonds in (b). In both cases, the broad diffraction by the liquid water is visible, in particular at low Q.

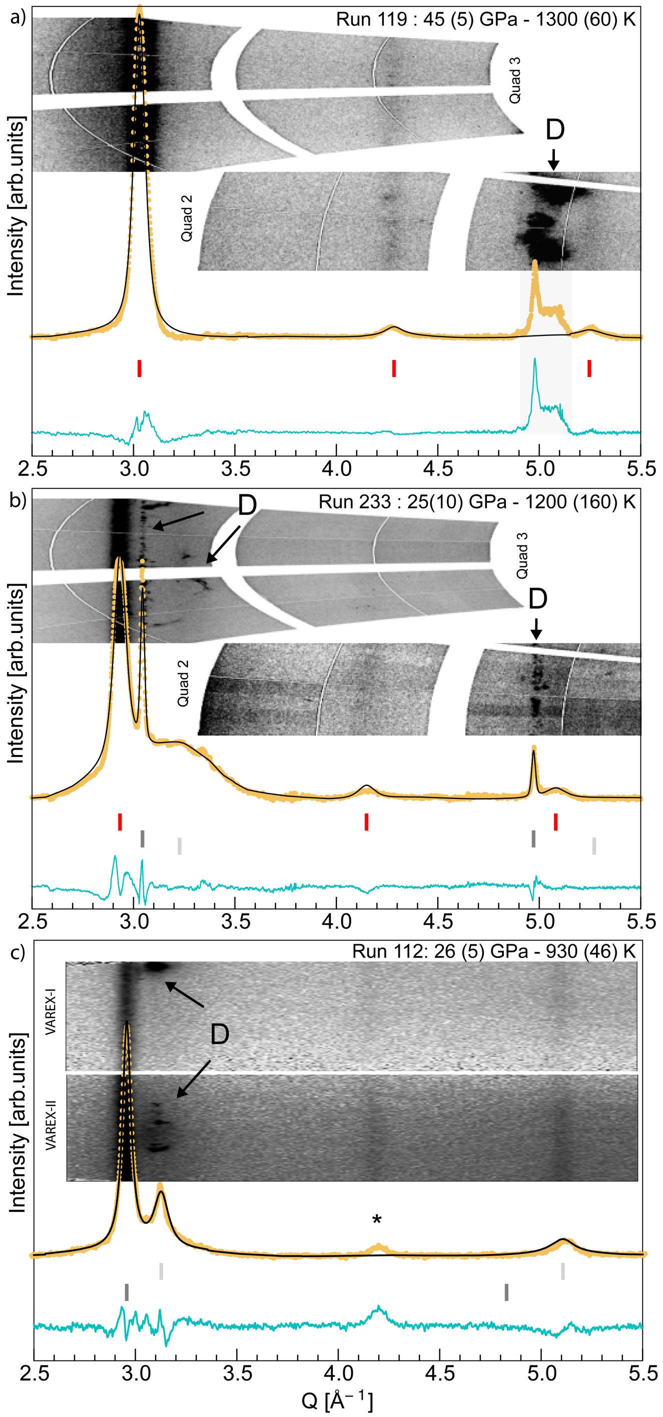

FIG. S7. Diffraction measurements from experiments probing the BCC structure, showing the 2D and the integrated XRD patterns collected during LCLS runs 119 (a) and 233 (b) and EuXFEL run 112 (c), respectively. In all panels, continuous black lines correspond to Le Bail fits of the experimental data (yellow points). Tick marks indicate the positions of Bragg reflections from the bcc phase (red) and diamond structures (gray and light gray). Panel (c) shows the same dataset as in Figure 3c of the main text, but refined using two diamond phases. This refinement highlights that the peak at 4.2 Å^{-1} originates from the BCC structure. In all panels the "D" indicates contributions from the diamonds windows and the cyan line shows the difference between the experimental and calculated intensities.

are presented in Fig. 3c. In all shots, two distinct textures are clearly visible: one arises from the powder sample and corresponds to a BCC structure with an $Im\bar{3}m$ space group, while the other, consisting of textured spots indicated by arrows, is attributed to diamonds with an $Fd\bar{3}m$ space group. At least three BCC peaks are observed, allowing for adequate refinement.
However, to remove any ambiguity, an alternative refinement was attempted considering two different diamond phases, as shown in Fig. S7c for shot 112. This alternative fit yields a lower-quality refinement, as the peak at 4.2 Å^{-1} (indicated by a star) remains unindexed. More-

over, one of the diamond phases exhibits an unphysical low density, implying negative pressures. Similar results are obtained when this approach is applied to the other two runs. These considerations exclude the possibility that the diffracted signal comes entirely from the diamonds and confirm the actual contribution from the compressed water.
The density values obtained for the BCC phases are presented in Table S1 and compared to experimental data from the literature in Fig. S17. Our BCC data points are in good agreement with those reported in earlier studies [9, 19–21]. In particular, they lie close to the equation of state (EOS) of superionic BCC (BCC-SI) proposed by Prakapenka *et al.* [20], which includes a thermal pressure correction, as well as to the BCC-SI point reported by Weck *et al.* [21]. This consistency suggests that the observed BCC lie within the superionic regime.

4.3. Observation of BCC + FCC coexistence

The coexistence of BCC and FCC phases was observed in four experimental shots (see Table S1). Fig. S8 presents 2D diffraction images along with details of the data refinement from experiments conducted at EuXFEL and LCLS under similar pressure-temperature conditions. The two sets of diffraction peaks are clearly distinguishable and can be indexed as BCC ($Im\bar{3}m$) and FCC ($Fd\bar{3}m$) structures. The derived densities for both phases are nearly identical within the experimental resolution, consistent with the results of the corresponding hydrodynamic simulations (Fig. S9) and previous experimental results [19, 20]. Furthermore, hydrodynamic simulations indicate relatively uniform pressures and temperatures throughout the sample, ruling out phase separation due to distinct thermodynamic conditions and supporting true phase coexistence. The repeated observation of this coexistence across four shots in two independent campaigns, under comparable conditions and with similar measured densities, reinforces this interpretation (Fig. S8c; Table S1). Furthermore, the deconvolution of the refinement profile for these shots (Fig. S8a,b) shows similar peak widths for the BCC and the FCC, indicating that both phases contribute coherently, with comparable degrees of crystallinity. This behaviour changes from the different peak widths observed for the faulted phases (see section 5).
For all shots, the measured densities are presented in Table S1 and compared to both experimental and theoretical results from the literature in Fig. S17b. The majority of our density values are in excellent agreement with those of FCC superionic at the same pressure, as reported by Weck *et al.* [21].These results further support the BCC–FCC phase boundary previously established under static compression conditions by Weck *et al.*

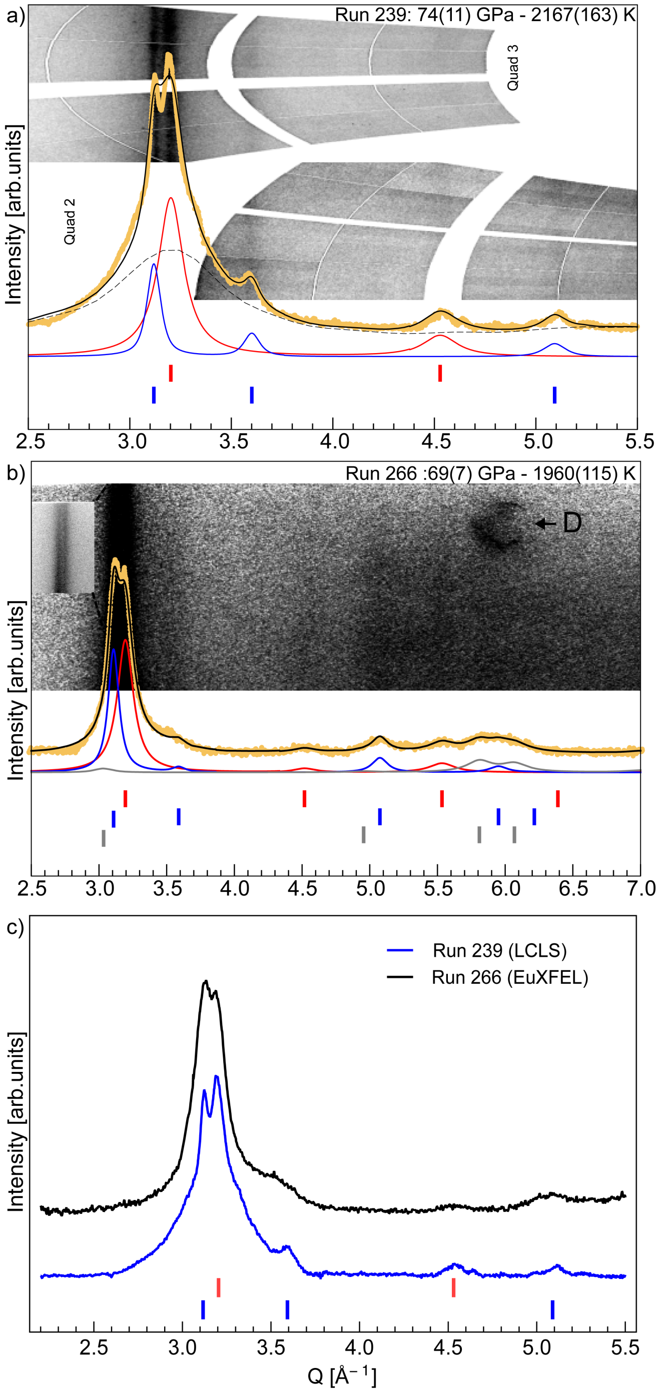

FIG. S8. Diffraction measurements from experiments probing the BCC-FCC phase coexistence. a) and b) display the 2D image and the integrated XRD data collected from LCLS Run 239 and EuXFEL Run 266, respectively. In both panels, the solid black lines correspond to the results of Le Bail fits applied to the experimental data (yellow dots). The red, blue and gray curves and tick represent the contributions of each phase and the Bragg reflections corresponding to the BCC, FCC, and diamond, respectively. The D indicates contributions from the diamonds windows. c) shows the comparison of the two integrated intensities from (a) and (b) indicating strong similarities.

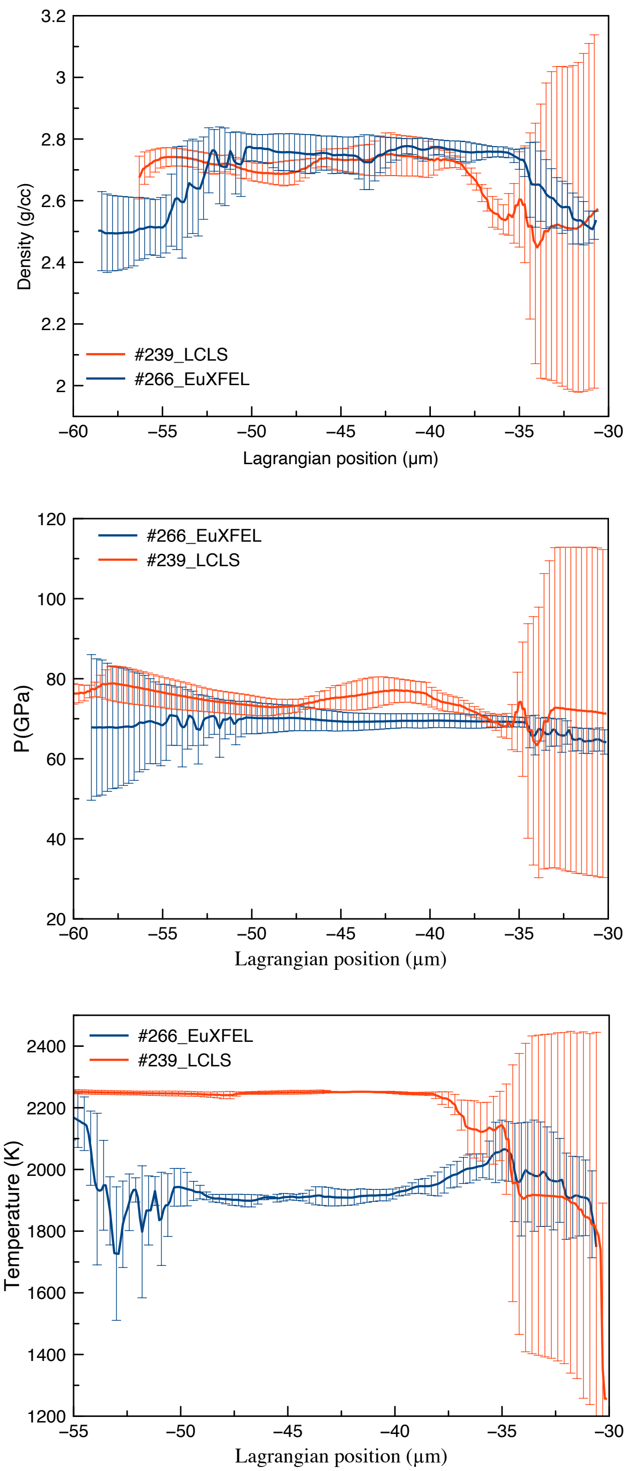

FIG. S9. Density (top) pressure (middle) and temperature (bottom) spatial profile across the water layer inferred from the optimised simulations for LCLS Run 239 and EuXFEL Run 266. For LCLS Run, only the colder part is presented (see section 3 and Fig. S4). Mean conditions and uncertainties are obtained from hydrodynamic simulations consistent within VISAR error bars.

5. REFINEMENT FOR STACKING FAULT

In all our data collected above ∼150 GPa, the diffraction patterns display asymmetric peak broadening that cannot be adequately reproduced by either a pure FCC structure (see section below) or a simple FCC + HCP two-phase model. An example of the Le Bail refinement using two distinct FCC and HCP phases is illustrated in Fig. S10. Given the large width of the HCP peaks in this case, we constrained the HCP lattice parameters to match the density of the FCC phase. This assumption allowed for a globally reasonable fit; however, several specific features favour an interpretation based on stacking disorder:

(i) Two-phase refinements consistently show asymmetry in the FWHM values of the FCC and HCP components, with the HCP showing strong broadening (see Fig. S10). This broadening points to increased local disorder. The increased broadening of the HCP peaks remains when performing individual pseudo-Voigt fits in Q-space for the representative reflections.
(ii) The FCC (200) peak systematically appears broadened and shifted toward lower Q values across all datasets, contrasting with the sharp and well-fitted profile observed at lower pressures (see Fig. S8). This behavior is a known signature of intrinsic stacking faults affecting the FCC lattice, as previously reported in the literature [22, 23] (see Fig. S11a).
(iii) The HCP (102) reflection is absent in all our diffraction patterns. This peak is known to be highly sensitive to deviations from ideal HCP stacking and tends to disappear in the presence of stacking disorder. A similar behavior has been observed in ZnS by Sebastian *et al.* [24], where specific reflections are suppressed due to stacking faults. In our case, as shown in the simulation (main text, Fig. 4c), the 102 peak only starts to emerge when the stacking sequence becomes predominantly HCP-like ($\alpha = 0.8$). This suggests that the HCP-like features observed in our data may originate from faulted stacking, rather than from a fully developed HCP phase.

Taken together, these observations suggest that stacking disorder provides a more consistent explanation for the diffraction features observed above 150 GPa than a simple two-phases FCC–HCP coexistence model.

To model the observed stacking disorder, we constructed a two-layer recursive stacking model using the FAULTS software package [18]. Each layer was structurally identical, but laterally shifted to reflect the typical stacking found in close-packed structures. The model was implemented in a hexagonal setting (see Fig. S11b), with a space group $R\bar{3}m$, and stacking disorder was introduced through probabilistic transitions between two layers. The stacking vectors for FCC and HCP sequences were defined as:

$$T_{\mathrm{fcc}} = \left(\tfrac{1}{3}a_h,, \tfrac{2}{3}a_h,, \tfrac{1}{3}c_h\right) \quad \text{and} \quad T_{\mathrm{hcp}} = \left(\tfrac{2}{3}a_h,, \tfrac{1}{3}a_h,, \tfrac{1}{3}c_h\right)$$

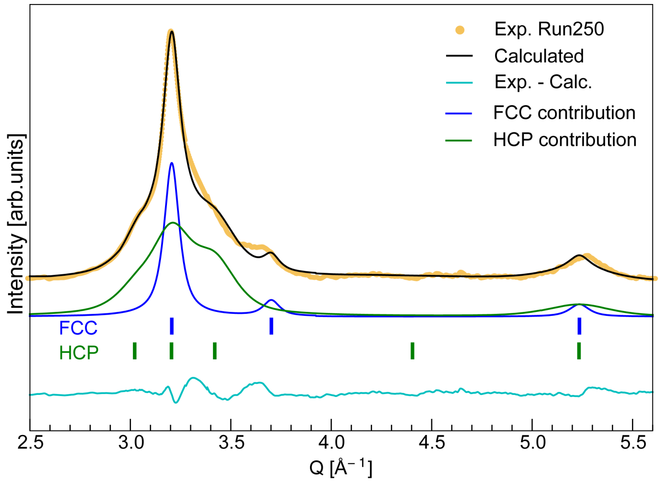

FIG. S10. Le Bail refinement (black line) of the data (yellow dots) considering the coexistence of FCC (blue) and HCP (green) phases, with the individual contribution of each phase. The cyan line represents the difference between the experimental and calculated intensities.

where the hexagonal lattice parameters a_h and c_h are related to the cubic parameter a_c by $a_h = \frac{\sqrt{2}}{2}a_c$ and $c_h = \sqrt{3}a_c$. Transitions of the type layer 1→1 and layer 2→2 were assigned the FCC vector, while transitions between layer 1→2 and layer 2→1 followed the HCP vector. The transition probabilities were denoted as α_{11}, α_{22} for same-layer transitions and α_{12}, α_{21} for cross-layer transitions. We assumed $\alpha_{11} = \alpha_{22}$ and $\alpha_{12} = \alpha_{21}$, with the additional constraint $\alpha_{12} = 1 - \alpha_{11}$. For simplicity, and consistently throughout this manuscript, we refer to α_{12} as α. By adjusting the value of α, we generated mixed stacking sequences with varying degrees of disorder. These probabilities were treated as free parameters during refinement, allowing for a quantitative estimation of the stacking disorder.

The influence of stacking disorder on the diffraction pattern was further explored through simulations, shown in Fig. 4c of the main text, where varying the stacking probability α highlights the gradual transition from ideal FCC stacking ($\alpha = 0$) to HCP-like stacking ($\alpha = 1$).

Importantly, since both layers are part of a single averaged hexagonal lattice, the model implicitly maintains a constant density across all stacking configurations.

The results of the refinements performed with FAULTS are summarized in Fig. S12 and Fig. 3a and 4b of the main text. This approach enabled a more realistic reproduction of the experimentally observed peak asymmetries, with estimated stacking fault probabilities ranging from 20% to 35%. However, EuXFEL diffractograms collected at wider angles clearly show an additional feature (Fig. 3a): the peak located at Q = 5.9 Å^{-1}, corresponding to the HCP (400) reflection, is not fully reproduced by the models. Similarly, the LCLS data, with better low-Q resolution (Fig. 4b), highlight subtle discrepancies between fit and experiment. In particular, small residual features are observed between Q = 3.4 Å^{-1}, which are not captured by the models. This suggests that the actual microstructure is more complex than a simple FCC–HCP layer alternation. Irregular stacking sequences, twins, or extrinsic stacking faults may be required to capture the full complexity of the observed structure, as proposed in previous works [22, 23]

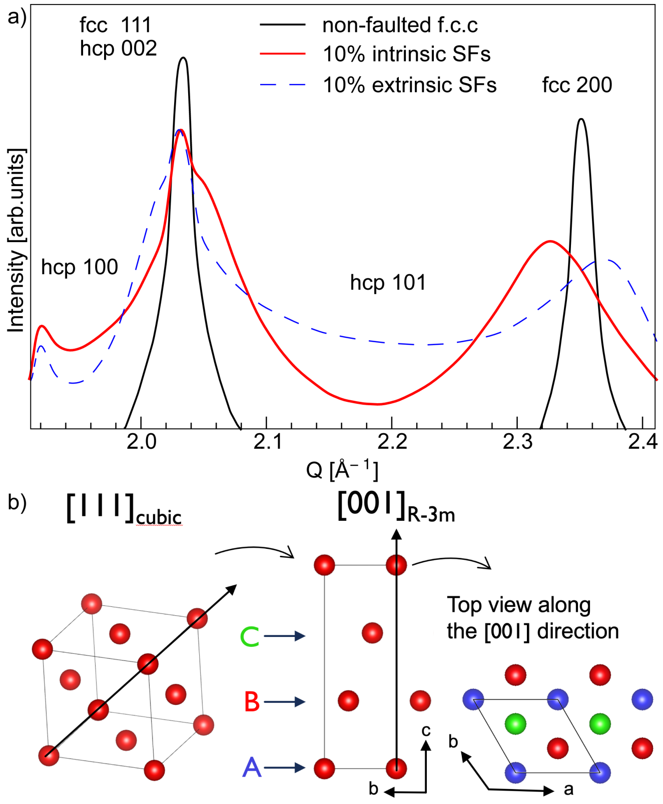

FIG. S11. a) Influence of stacking faults on peak position, broadening, and asymmetry in simulated XRD patterns. Simulations were performed using DIFFaX and adapted from [22, 23]. The black line corresponds to a non-faulted FCC structure, the red line shows a structure containing 10% intrinsic stacking faults, and the blue line represents 10% extrinsic stacking faults. Stacking faults lead to asymmetric broadening and shifts in both the (111) and (200) reflections, illustrating the sensitivity of peak shapes to microstructural disorder. b) Representation of closely packed FCC in the hexagonal coordinate system.

The measured FCC/HCP densities are summarized in Table S1 and compared to literature data in Fig. S17b. This includes experimental results obtained under dynamic compression, static compression as well as theoretical predictions [20, 21, 25, 26]. Overall, our measurements fall within the range defined by these studies, providing additional support for the consistency of our structural interpretation.

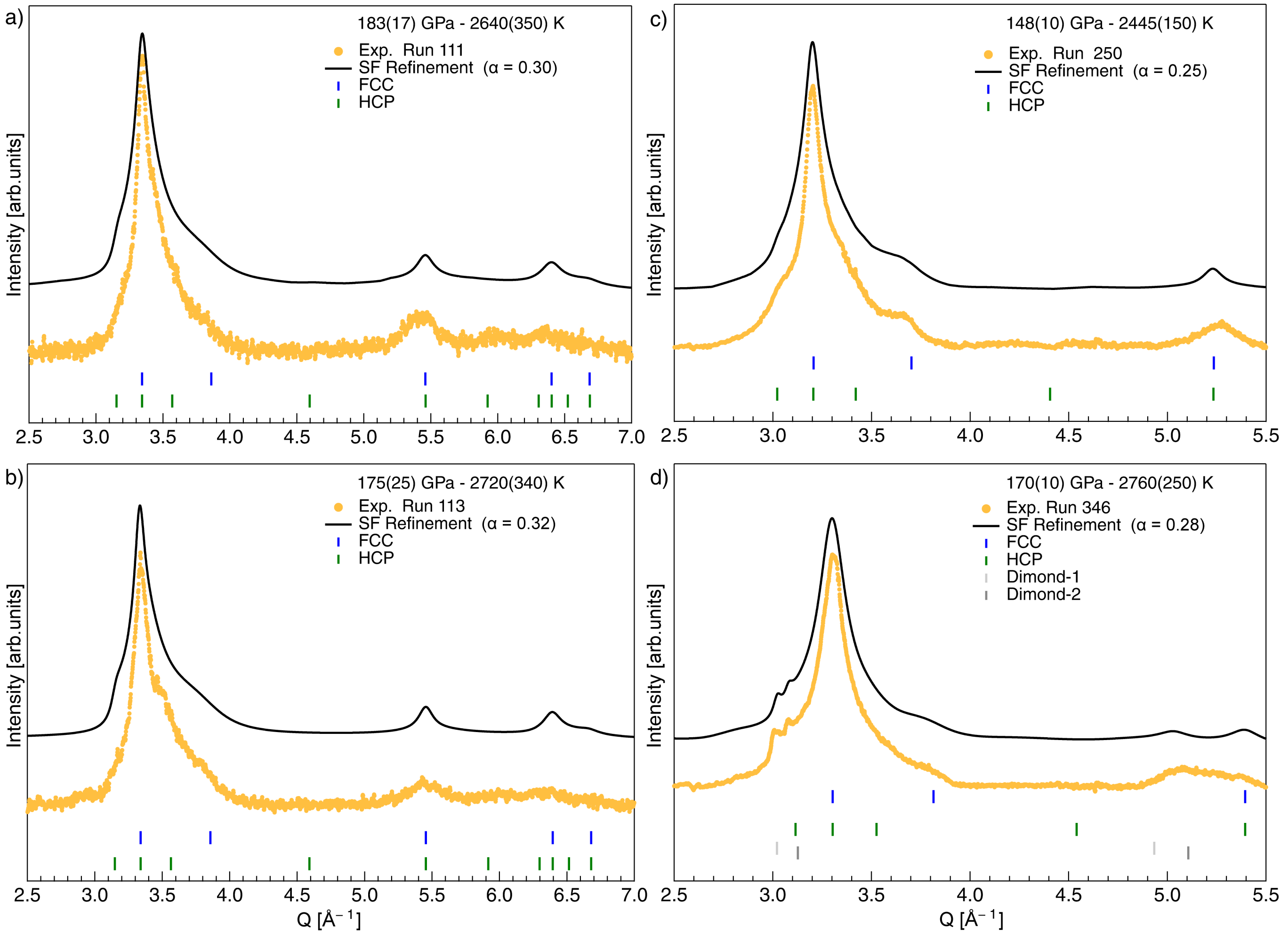

FIG. S12. Refinement of our diffraction data demonstrating the presence of stacking disorder. Panels (a) and (b) respectively show EuXFEL runs 111 (as in Fig. 3a) and 113, while panels (c) and (d) present LCLS runs 250 (as in Fig. 4b) and 346. The solid black line represents the result of the stacking faults refinement. The vertical lines indicate the expected Bragg peaks for FCC (blue), HCP (green), and diamonds (gray). For the LCLS datasets, a liquid phase contribution is often present. This contribution was modeled and included as part of the background during refinement. In cases where diamond peaks were visible, they were first refined separately using FullProf in a three-phase (FCC+HCP+D) model, allowing us to generate a reference profile for the diamond contribution. This profile was then imported into the FAULTS refinement as a fixed background component.

6. RULING OUT OTHER POSSIBLE INTERPRETATIONS OF THE SF DATA

In order to corroborate our interpretation of the high-pressure diffraction patterns in terms of stacking faults, we evaluate several alternative explanations, including a pure FCC phase, a coexistence of BCC and FCC phases, contributions from diamond or liquid phases and grain-size effects. As outlined below, none of these alternatives provide a satisfactory match of our data, thus reinforcing the validity of the stacking fault scenario. The presented analysis is based on the archetypal EuXFEL Run 111, but the same considerations can be transferred to the whole set of high-pressure data.

6.1. FCC only phase

Fig. S13 shows the refinement was performed by assuming an FCC structure with a space group $Fm\bar{3}m$. This refinement revealed that some peaks, particularly the most intense one, can be indexed by an FCC structure with a lattice parameter (a = 3.255(1) Å). However, several distinctive features remain poorly fitted. Notably the broad peaks at 3.2 and 3.6 Å^{-1} in Q are clearly visible in the experimental data (see the inset in Fig. S13), but cannot be explained by using the sole FCC structure.

6.2. Mixture of FCC + BCC structures

Another possibility we tested concerns the coexistence of BCC and FCC phase, as we observe at lower conditions.

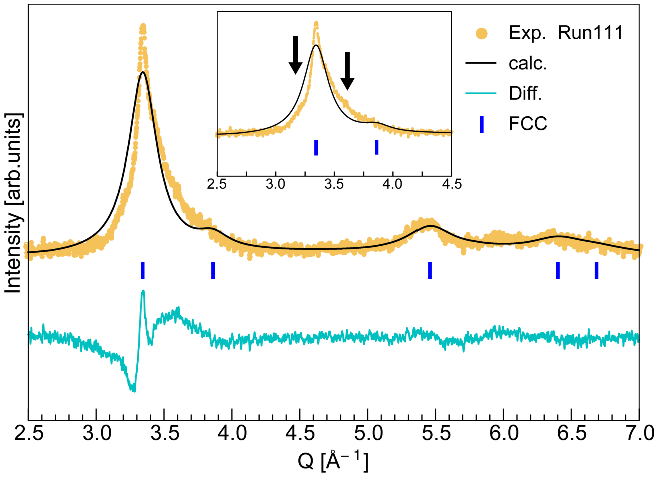

FIG. S13. Refinement of experimental data assuming an FCC phase. An inset shows a zoom around the FCC (111) reflection, highlighting additional contributions near 3.2 and 3.6 Å^{-1} that are not accounted by the FCC structure. Yellow dots, cyan and black lines as in the other figures.

We first assumed that the two phases have the same density, as observed at lower pressures. Therefore, we set the density of the BCC phase at 3.47 g/cm^3, which corresponds to a lattice parameter of 2.583(1) Å. As shown in, Fig. S14a, the fitting model obtained is not satisfactory, as this combination does not explain the peak observed at 3.6 Å^{-1}.

Secondly, we considered the existence of a pressure gradient in the sample, which would imply a density difference between the two phases. This approach would allow us to match the (110) reflection of the BCC phase with the shoulder observed at 3.2 Å^{-1}, which would give a density of 2.76 g/cm^3. However, as illustrated in Fig. S14b this assumption still does not provide a satisfactory fit, as the peak at 3.6 Å^{-1} cannot be well reproduced. Moreover such pressure gradients are not predicted from the hydrodynamic simulations (see Fig. S4d).

6.3. Diamond contribution

We also considered the possibility that these features would be linked to the diamond ablator and/or the diamond window. We therefore tried to refine the diffracted data by constraining the diamond density to 4.036140

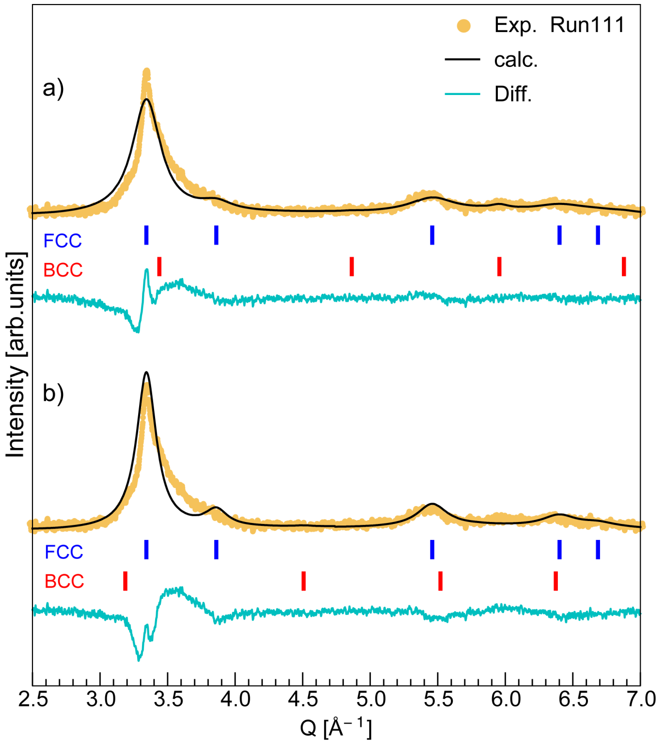

FIG. S14. Refinement of high-pressure experimental data using a combined FCC + BCC model. (a) Assuming both phases have the same density (3.47 g/cm^3). (b) Assuming a pressure gradient, with the BCC (110) peak shifted to match the observed peak at 3.2 Å^{-1}. Yellow dots, cyan and black lines as in the other figures.

g/cm^{3}, so that the (111) reflection corresponded to the peak located at 3.2 Å^{-1}. However, the result shows that this phase combination still does not explain the observed peaks at 3.6 Å^{-1}. The inclusion of an additional diamond contribution at a higher density could be used to explain such peak. This approach, though, would result in an extremely high density for the second diamond, at $\sim$ 5.6 g/cm^{3}, corresponding to a pressure of $\sim$ 500 GPa, far beyond our reachable conditions.
The contribution of diamonds can be completely ruled out, as they cannot be observed on the image plates. In fact, rapid and intense compression transforms diamonds into highly textured polycrystals, as illustrated in Fig. S7 and Fig. S8. However, by examining the image plates of the EuXFEL Run 111 and the LCLS Run 250 (shot in which this phase is also observed), the diffraction signals appear relatively homogeneous, with no noticeable difference in texture. This confirms that these "extra peaks" do not originate from diamond.

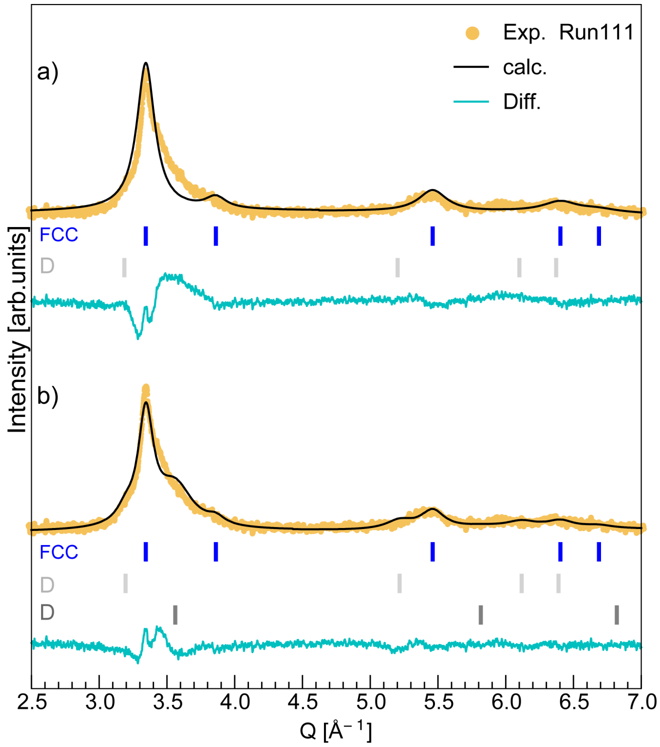

FIG. S15. Refinement of high-pressure experimental data using a combined FCC + diamond model. (a) Using a single diamond phase and constraining the density to 4.036 g/cm^{3}, aligning the diamond (111) reflection with the peak observed at 3.2 Å^{-1}. (b) Using two diamond phases with different densities. Yellow dots, cyan and black lines as in the other figures.

6.4. Liquid Contribution

Broadening of the peaks could also result from the liquid background coming from a molten part of the target. To assess its impact on the diffraction, we considered the contribution of a simulated liquid structure. The liquid structure was calculated at 2400 K and 135 GPa using the classical molecular dynamics code LAMMPS [27] together with the n2p2 module [28] and the DFT-based water machine learning potential developed by Cheng *et al.* [29]. The simulation contained 512 water molecules and started from a molecular liquid structure. The NpT simulation was run for 400000 time steps with a time step size of 0.25 fs.

The results of this simulation, together with a fit that combines the FCC and the liquid, are shown in Fig. S16. From the fitting process, it is clear that the liquid backgrounds and FCC phase alone could not adequately explain the diffraction patterns experimentally observed for these higher intensity shots.

6.5. Grain size broadening

Another possible contribution that could cause diffraction peak broadening is the grain size effect. To evaluate

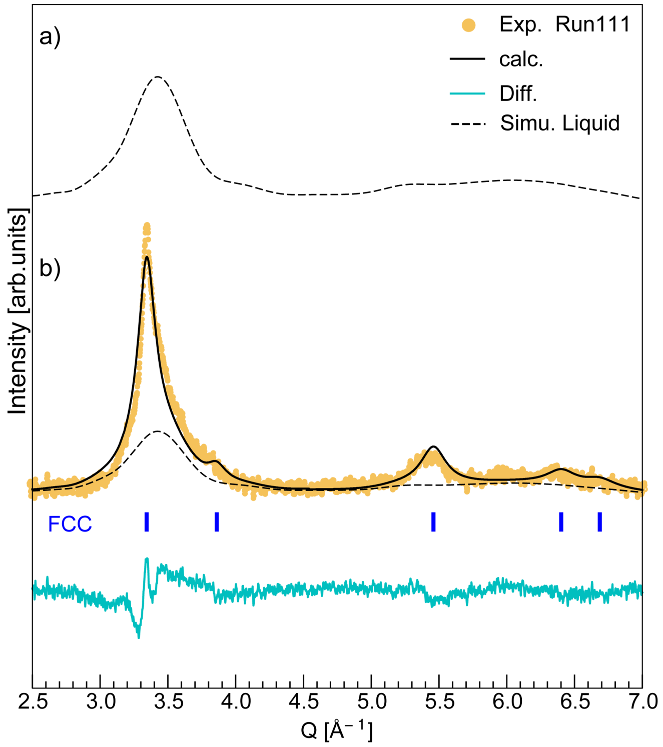

FIG. S16. a) Simulation of the liquid structure at 2400 K and 135 GPa (dotted black line). b) Refinement of high-pressure experimental data using a combined FCC phase and liquid contribution. Yellow dots, cyan and black lines as in the other figures.

this contribution, we applied the Williamson–Hall (WH) [30] to two datasets: one collected at ∼ 26 GPa (Run 112) and the other at ∼ 183 GPa (Run 111) (see Table S1).

This method allows us to distinguish peak broadening caused by finite crystallite size from that induced by microstrain. The equation used for this analysis is:

$$\beta \cos\theta = \frac{K\lambda}{D} + 4\varepsilon \sin\theta \tag{1}$$

where β represents the full width at half maximum (FWHM) corrected for instrumental broadening, θ is the Bragg angle, K is the shape factor (typically close to 0.9), λ is the wavelength of the X-rays (0.688 Å), D is the average crystallite size, and ε is the microstrain.

Plotting $\beta \cos\theta$ as a function of $\sin\theta$ yields a straight line, whose slope corresponds to 4ε, and whose intercept is related to the crystallite size through the term $\frac{K\lambda}{D}$.

The WH analysis performed on the Run 112 data indicates an average crystallite size of $D \approx 44$ nm and a microstrain of $\varepsilon \approx 0.07\%$. For comparison, the analysis of Run 111 yielded a crystallite size of $D \approx 33$ nm, but with a significantly higher microstrain of $\varepsilon \approx 2.2\%$

These results indicate that the crystallite size remains relatively constant under both conditions, consistently within the nanometre range, comparable to the grain sizes reported for SiO_2 under shock compression [31]. In contrast, the microstrain increases significantly at high pressure, indicating that the peak broadening observed is primarily driven by microstructural changes. This evolution reflects the buildup of internal stresses, likely associated with increased crystallographic disorder.

7. H_2O HIGH-PRESSURE DIAGRAM

To provide a more comprehensive picture, in Supplementary Fig. S18, we show our data together with earlier X-ray diffraction studies of water ice, under both dynamic and static compression, alongside theoretical phase boundaries from the literature, and conditions where the liquid phase was observed in our hydrodynamic simulations.

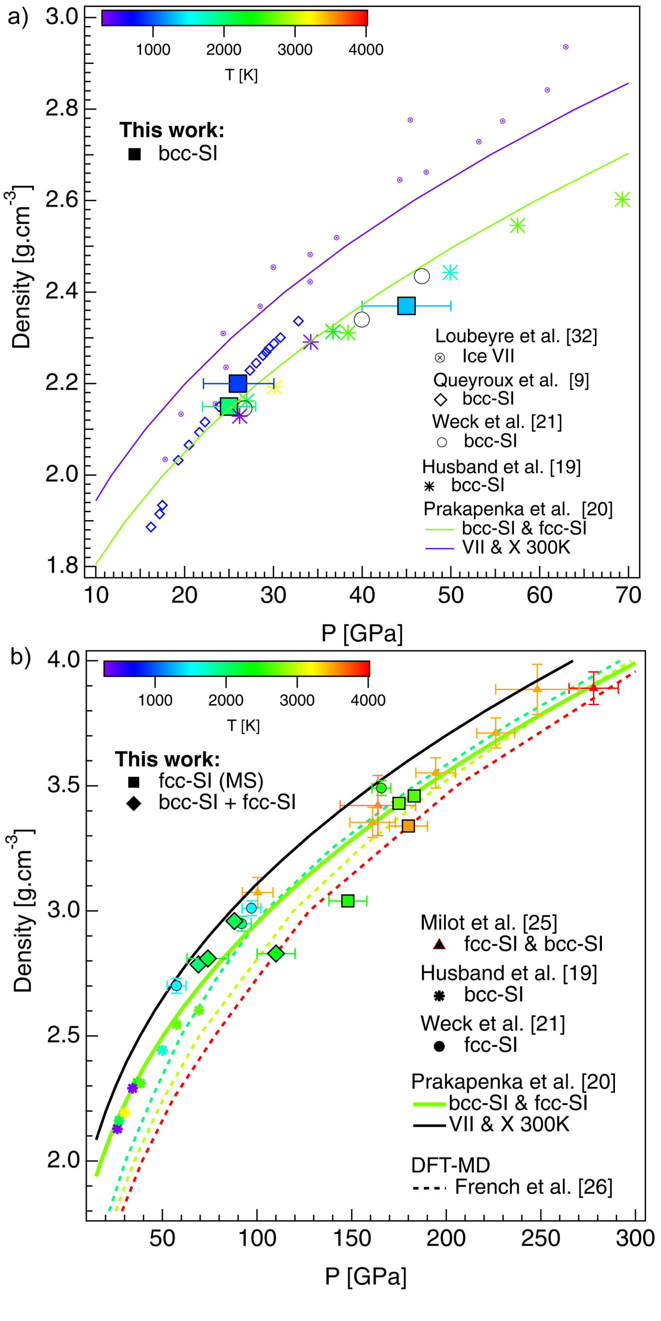

FIG. S17. Compare the experimental densities documented here with those from previously published theoretical and experimental studies [9, 19–21, 25, 32]. Error bars on our data represent the variation among simulations consistent with the VISAR uncertainty. a) Presents all the data at low pressure, where a simple BCC structure is observed. b) Shows the densities for the mixed phases: BCC + FCC and the FCC/HCP mixed stacking.

Run number	Phase	ρ(bcc) [g/cm^3]	ρ(fcc) [g/cm^3]	$\rho_{sim}(H_2O)$ [g/cm^3]	P [GPa]	T [K]	E [J]	t_{probe} [ns]
112	BCC	2.207(2)	-	2.07(5)	26(5)	930(46)	17.5	12
**119**	BCC	2.371(8)	-	2.3(1)	45 (5)	1300(60)	34.9	9
**233**	BCC	2.153(9)	-	2.1(1)	25(10)	1200(160)	37	12
**239**	BCC+FCC	2.799(3)	2.815(2)	2.67(8)	74(11)	2167(163)	34	13.5
**245**	BCC+FCC	2.844(4)	2.821(2)	2.99(2)	103(6)	2415(155)	34	14
248	BCC+FCC	3.02(2)	2.96(1)	2.89(14)	88(12)	2099(124)	22.2	10.5
266	BCC+FCC	2.780(7)	2.786(5)	2.68(1)	69(7)	1960(115)	22	14
111	FCC/HCP	-	3.46(3)	3.51(17)	183(17)	2640(350)	28	10
113	FCC/HCP	-	3.43(2)	3.39(12)	175(25)	2720(340)	33.3	10
**250**	FCC/HCP	-	3.04(3)	3.23(5)	148(10)	2445(150)	57	12.5
**346**	FCC/HCP	-	3.34(3)	3.36(6)	170(10)	2760(250)	59	11

TABLE S1. Summary of results for all runs collected in both experiments at EuXFEL and LCLS. The runs collected at LCLS are highlighted in bold. Error bars on density represent the variation among simulations consistent with the VISAR uncertainty.

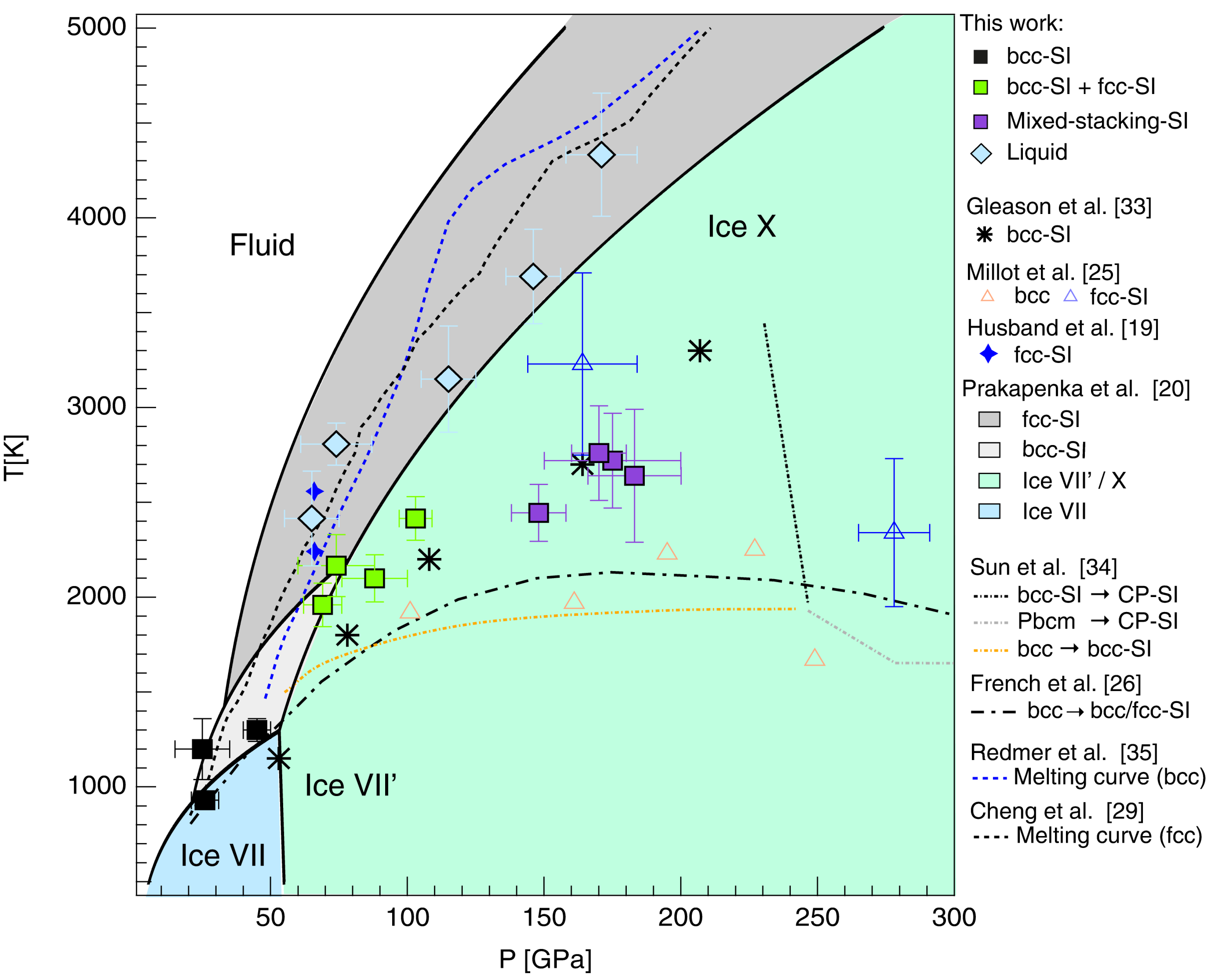

FIG. S18. P-T diagram of H_2O below 300 GPa including additional experimental data showing different phase stability domains. The calculated pressure–temperature conditions from our data (square), also include the conditions where the liquid phase was observed in our hydrodynamic simulations (diamond). Error bars on P–T conditions represent the variation among simulations consistent with the VISAR uncertainty. Measured P/T conditions from previous X-ray diffraction experiments are also shown.Data from dynamic compression are taken from Millot *et al.* [25], Gleason *et al.* [33], and Husband *et al.* [19], while data from static compression are taken from Prakapenka *et al.* [20]. Colored areas correspond to the phase diagram proposed in ref. [20], as indicated in the figure legend. Published theoretical phase boundaries from French *et al.* [26], Cheng *et al.* [29], and Sun *et al.* [34], Redmer *et al.* [35], are also included for comparison.

[1] P. Celliers, G. Collins, L. Da Silva, D. Gold, and R. Cauble, Accurate measurement of laser-driven shock trajectories with velocity interferometry, Applied Physics Letters **73**, 1320 (1998).

[2] K. Katagiri, N. Ozaki, K. Miyanishi, N. Kamimura, Y. Umeda, T. Sano, T. Sekine, and R. Kodama, Optical properties of shock-compressed diamond up to 550 gpa, Phys. Rev. B **101**, 184106 (2020).

[3] S. Bardy, B. Aubert, T. Bergara, L. Berthe, P. Combis, D. Hébert, E. Lescoute, Y. Rouchausse, and L. Videau, Development of a numerical code for laser-induced shock waves applications, Optics and Laser Technology **124**, 105983 (2020).

[4] R. S. McWilliams, J. H. Eggert, D. G. Hicks, D. K. Bradley, P. M. Celliers, D. K. Spaulding, T. R. Boehly, G. W. Collins, and R. Jeanloz, Strength effects in diamond under shock compression from 0.1 to 1 tpa, Phys. Rev. B **81**, 014111 (2010).

[5] J. Haldemann, Y. Alibert, C. Mordasini, and W. Benz, Aqua: a collection of h2o equations of state for planetary models, Astronomy & Astrophysics **A105**, 18 (2020).

[6] M. French and R. Redmer, Construction of a thermodynamic potential for the water ices vii and x, Physical Review B **91**, 014308 (2015).

[7] S. Mazevet, A. Licari, G. Chabrier, and A. Y. Potekhin, Ab initio based equation of state of dense water for planetary and exoplanetary modeling, Astronomy & Astrophysics **621**, A128 (2019).

[8] J.-A. Hernandez and R. Caracas, Superionic-superionic phase transitions in body-centered cubic h_2O ice, Phys. Rev. Lett. **117**, 135503 (2016).

[9] J.-A. Queyroux, J.-A. Hernandez, G. Weck, S. Ninet, T. Plisson, S. Klotz, G. Garbarino, N. Guignot, M. Mezouar, M. Hanfland, J.-P. Itié, and F. Datchi, Melting curve and isostructural solid transition in superionic ice, Phys. Rev. Lett. **125**, 195501 (2020).

[10] S. P. Lyon and J. D. Johnson, "sesame: The los alamos national laboratory equation of state database", Los Alamos National Laboratory Report **LA-UR-92-3407** (1992).

[11] M. French, T. R. Mattsson, N. Nettelmann, and R. Redmer, Equation of state and phase diagram of water at ultrahigh pressures as in planetary interiors, Physical Review B **79**, 054107 (2009).

[12] M. Bethkenhagen, E. R. Meyer, S. Hamel, N. Nettelmann, M. French, L. Scheibe, C. Ticknor, L. A. Collins, J. D. Kress, J. J. Fortney, and R. Redmer, Planetary ices and the linear mixing approximation, The Astrophysical Journal **848**, 67 (2017).

[13] D. H. Dolan, *Foundations of VISAR analysis.*, Tech. Rep. (Sandia National Laboratories (SNL), Albuquerque, NM, and Livermore, CA (United States), 2006).

[14] A. Dewaele, J. H. Eggert, P. Loubeyre, and R. Le Toullec, Measurement of refractive index and equation of state in dense he, h_2, h_2O, and ne under high pressure in a diamond anvil cell, Phys. Rev. B **67**, 094112 (2003).

[15] C. Prescher and V. B. Prakapenka, Dioptas: a program for reduction of two-dimensional x-ray diffraction data and data exploration, High Pressure Research **35**, 223 (2015), https://doi.org/10.1080/08957959.2015.1059835.

[16] J. Rodríguez-Carvajal, Recent advances in magnetic structure determination by neutron powder diffraction, Physica B: Condensed Matter **192**, 55 (1993).

[17] M. Treacy, J. Newsam, and M. Deem, A general recursion method for calculating diffracted intensities from crystals containing planar faults, Proceedings of the Royal Society of London. Series A: Mathematical and Physical Sciences **433**, 499 (1991).

[18] M. Casas-Cabanas, M. Reynaud, J. Rikarte, P. Horbach, and J. Rodriguez-Carvajal, Faults: a program for refinement of structures with extended defects, Journal of Applied Crystallography **49**, 2259 (2016).

[19] R. J. Husband, H. P. Liermann, J. D. McHardy, R. S. McWilliams, A. F. Goncharov, V. B. Prakapenka, E. Edmund, S. Chariton, Z. Konôpková, C. Strohm, C. Sanchez-Valle, M. Frost, L. Andriambariarijaona, K. Appel, C. Baehtz, O. B. Ball, R. Briggs, J. Buchen, V. Cerantola, J. Choi, A. L. Coleman, H. Cynn, A. Dwivedi, H. Graafsma, H. Hwang, E. Koemets, T. Laurus, Y. Lee, X. Li, H. Marquardt, A. Mondal, M. Nakatsutsumi, S. Ninet, E. Pace, C. Pepin, C. Prescher, S. Stern, J. Sztuk-Dambietz, U. Zastrau, and M. I. McMahon, Phase transition kinetics of superionic h2o ice phases revealed by megahertz x-ray free-electron laser-heating experiments, Nature Communications **15**, 8256 (2024).

[20] V. B. Prakapenka, N. Holtgrewe, S. S. Lobanov, and A. F. Goncharov, Structure and properties of two superionic ice phases, Nature Physics **17**, 1233 (2021).

[21] G. Weck, J.-A. Queyroux, S. Ninet, F. Datchi, M. Mezouar, and P. Loubeyre, Evidence and stability field of fcc superionic water ice using static compression, Phys. Rev. Lett. **128**, 165701 (2022).

[22] L. B. Fletcher, A. L. Levitan, E. E. McBride, J. B. Kim, E. P. Alves, A. Aquila, M. Frost, S. Goede, G. King, T. J. Lane, M. Liang, M. J. MacDonald, B. K. Ofori-Okai, C. Schönwälder, P. Sun, J. B. Hastings, S. Boutet, and S. H. Glenzer, X-ray diffraction of metastable structures from supercooled liquid hydrogen, Scientific Reports **14**, 17283 (2024).

[23] S. Martin, C. Ullrich, D. Šimek, U. Martin, and D. Rafaja, Stacking fault model of ε-martensite and its *DIFFaX* implementation, Journal of Applied Crystallography **44**, 779 (2011).

[24] M. T. Sebastian and P. Krishna, X-ray diffraction effects from randomly twinned f.c.c. crystals undergoing transformation to the h.c.p. phase, Acta Crystallographica Section B **43**, 409 (1987).

[25] M. Millot, F. Coppari, J. R. Rygg, A. Correa Barrios, S. Hamel, D. C. Swift, and J. H. Eggert, Nanosecond x-ray diffraction of shock-compressed superionic water ice, Nature **569**, 251 (2019).

[26] M. French, M. Desjarlais, and R. Redmer, *Ab initio* calculation of thermodynamic potentials and entropies for superionic water, Physical Review E **93**, 022140 (2016).

[27] A. P. Thompson, H. M. Aktulga, R. Berger, D. S. Bolintineanu, W. M. Brown, P. S. Crozier, P. J. in 't Veld, A. Kohlmeyer, S. G. Moore, T. D. Nguyen, R. Shan, M. J. Stevens, J. Tranchida, C. Trott, and S. J. Plimpton, Lammps - a flexible simulation tool for particle-based materials modeling at the atomic, meso, and continuum

scales, Computer Physics Communications **271**, 108171 (2022).

[28] A. Singraber, J. Behler, and C. Dellago, Library-based lammps implementation of high-dimensional neural network potentials, Journal of Chemical Theory and Computation **15**, 1827 (2019).

[29] B. Cheng, M. Bethkenhagen, C. J. Pickard, and S. Hamel, Phase behaviours of superionic water at planetary conditions, Nature Physics **17**, 1228 (2021).

[30] G. Williamson and W. Hall, X-ray line broadening from filed aluminium and wolfram, Acta metallurgica **1**, 22 (1953).

[31] A. E. Gleason, C. A. Bolme, H. J. Lee, B. Nagler, E. Galtier, D. Milathianaki, J. Hawreliak, R. G. Kraus, J. H. Eggert, D. E. Fratanduono, G. W. Collins, R. Sandberg, W. Yang, and W. L. Mao, Ultrafast visualization of crystallization and grain growth in shock-compressed sio2, Nature Communications **6**, 8191 (2015).

[32] P. Loubeyre, R. LeToullec, E. Wolanin, M. Hanfland, and D. Hausermann, Modulated phases and proton centring in ice observed by x-ray diffraction up to 170?gpa, Nature **397**, 503 (1999).

[33] A. E. Gleason, D. R. Rittman, C. A. Bolme, E. Galtier, H. J. Lee, E. Granados, S. Ali, A. Lazicki, D. Swift, P. Celliers, B. Militzer, S. Stanley, and W. L. Mao, Dynamic compression of water to conditions in ice giant interiors, Scientific Reports **12**, 715 (2022).

[34] J. Sun, B. K. Clark, S. Torquato, and R. Car, The phase diagram of high-pressure superionic ice, Nature Communications **6**, 8156 (2015).

[35] R. Redmer, T. R. Mattsson, N. Nettelmann, and M. French, The phase diagram of water and the magnetic fields of uranus and neptune, Icarus **211**, 798 (2011).
